# Cerebrospinal fluid NCAM-1 concentration is associated with neurodevelopmental outcome in post-hemorrhagic hydrocephalus of prematurity

**DOI:** 10.1371/journal.pone.0247749

**Published:** 2021-03-10

**Authors:** David D. Limbrick, Diego M. Morales, Chevis N. Shannon, John C. Wellons, Abhaya V. Kulkarni, Jessica S. Alvey, Ron W. Reeder, Volker Freimann, Richard Holubkov, Jay K. Riva-Cambrin, William E. Whitehead, Curtis J. Rozzelle, Mandeep Tamber, W. Jerry Oakes, James M. Drake, Ian F. Pollack, Robert P. Naftel, Terrie E. Inder, John R. Kestle, for the Hydrocephalus Clinical Research Network

**Affiliations:** 1 Department of Neurosurgery, Washington University St. Louis, St. Louis, MO, United States of America; 2 Department of Neurosurgery, Vanderbilt University Medical Center, Nashville, TN, United States of America; 3 Department of Neurosurgery, University of Toronto, Toronto, ON, Canada; 4 Data Coordinating Center, University of Utah, Salt Lake City, UT, United States of America; 5 Division of Neurosurgery, University of Calgary, Calgary, AB, Canada; 6 Department of Neurosurgery, Baylor College of Medicine, Houston, TX, United States of America; 7 Department of Neurosurgery, University of Alabama–Birmingham, Birmingham, AL, United States of America; 8 Department of Surgery, University of British Columbia, Vancouver, BC, Canada; 9 Department of Neurosurgery, University of Pittsburgh Medical Center, Pittsburgh, PA, United States of America; 10 Department of Pediatric Newborn Medicine, Harvard Medical School, Boston, MA, United States of America; 11 Department of Neurosurgery, University of Utah, Salt Lake City, UT, United States of America; Western University, CANADA

## Abstract

**Objective:**

Efforts directed at mitigating neurological disability in preterm infants with intraventricular hemorrhage (IVH) and post hemorrhagic hydrocephalus (PHH) are limited by a dearth of quantifiable metrics capable of predicting long-term outcome. The objective of this study was to examine the relationships between candidate cerebrospinal fluid (CSF) biomarkers of PHH and neurodevelopmental outcomes in infants undergoing neurosurgical treatment for PHH.

**Study design:**

Preterm infants with PHH were enrolled across the Hydrocephalus Clinical Research Network. CSF samples were collected at the time of temporizing neurosurgical procedure (n = 98). Amyloid precursor protein (APP), L1CAM, NCAM-1, and total protein (TP) were compared in PHH versus control CSF. Fifty-four of these PHH subjects underwent Bayley Scales of Infant Development-III (Bayley-III) testing at 15–30 months corrected age. Controlling for false discovery rate (FDR) and adjusting for post-menstrual age (PMA) and IVH grade, Pearson’s partial correlation coefficients were used to examine relationships between CSF proteins and Bayley-III composite cognitive, language, and motor scores.

**Results:**

CSF APP, L1CAM, NCAM-1, and TP were elevated in PHH over control at temporizing surgery. CSF NCAM-1 was associated with Bayley-III motor score (R = -0.422, p = 0.007, FDR Q = 0.089), with modest relationships noted with cognition (R = -0.335, p = 0.030, FDR Q = 0.182) and language (R = -0.314, p = 0.048, FDR Q = 0.194) scores. No relationships were observed between CSF APP, L1CAM, or TP and Bayley-III scores. FOHR at the time of temporization did not correlate with Bayley-III scores, though trends were observed with Bayley-III motor (p = 0.0647 and R = -0.2912) and cognitive scores (p = 0.0506 and R = -0.2966).

**Conclusion:**

CSF NCAM-1 was associated with neurodevelopment in this multi-institutional PHH cohort. This is the first report relating a specific CSF protein, NCAM-1, to neurodevelopment in PHH. Future work will further investigate a possible role for NCAM-1 as a biomarker of PHH-associated neurological disability.

## Introduction

Post-hemorrhagic hydrocephalus (PHH) of prematurity is the most common cause of pediatric hydrocephalus in North America [1, 2] and requires complex, lifelong neurosurgical care. Moreover, PHH carries a substantial risk of neurological disability, with 85% of affected children experiencing cognitive deficits and 70% suffering motor deficits [3–5]. Despite these risks, there is no consensus regarding the treatment of PHH [6]. Investigators in the field continue to explore management strategies to mitigate neurological disability [7–9], though such efforts have been limited in large part due to a dearth of quantifiable PHH metrics capable of predicting post-surgical neurodevelopmental outcomes.

Neurosurgical management strategies for PHH in preterm infants may be broadly considered as temporizing procedures [ventricular reservoirs (RES), ventriculo-subgaleal shunts (VSGS)] and permanent procedures [cerebrospinal fluid (CSF) shunts, endoscopic third ventriculostomy (ETV) +/- choroid plexus cauterization (CPC)]. There is wide variability in clinical pediatric neurosurgical practice in the timing and use of these procedures, with few Level I studies [10–17] and most other studies (Level II-III) focusing on surgical parameters rather than neurodevelopmental outcomes [1, 6]. More recently, there has been increased attention on neurodevelopment, though findings regarding the role of surgical intervention on outcomes are mixed [7–9, 18].

Cerebrospinal fluid biomarkers have been shown to accurately reflect real-time pathophysiology in a number of neurological diseases [19–25], including hydrocephalus [26]. While many CSF proteins have been investigated as candidate biomarkers of PHH [26, 27], a recent data-driven proteomics approach, based on strength of statistical association and effect size, implicated the protein mediators of neurodevelopment [28] and ventricular zone biology [29] amyloid precursor protein (APP), neural cell adhesion molecule 1 (NCAM-1), and L1 cell adhesion molecule (L1CAM) as candidate CSF biomarkers of neurodevelopment/neural injury in PHH. Subsequent studies have further supported the relationships of CSF APP, NCAM-1, and L1CAM to ventricular size and intracranial pressure, surrogates of PHH severity, and the responsiveness of these CSF biomarkers to neurosurgical treatment [30, 31].

The objective of the current study was to investigate the associations between the levels of CSF APP, NCAM-1, and L1CAM at the time of temporizing neurosurgical procedure, *ie* prior to PHH treatment, and neurodevelopmental outcome in PHH. To this end, CSF samples and clinical data were collected from consenting patients at participating Hydrocephalus Clinical Research Network (HCRN) sites, and CSF APP, NCAM-1, and L1CAM levels were related to neurodevelopmental outcomes measured using the Bayley Scales of Infant Development-III (Bayley-III) obtained in these same subjects at 15–30 months corrected age.

## Materials and methods

### Research subjects

At the inception of the CSF Biomarkers of PHH study, the HCRN comprised 7 centers, all of which participated in this study: Vanderbilt University/Monroe Carell Jr. Children’s Hospital, University of Alabama-Birmingham/Children’s of Alabama, University of Utah/Primary Children’s Hospital, University of Toronto/The Hospital for Sick Children, Baylor University/Texas Children’s Hospital, University of Pittsburgh/Children’s Hospital of Pittsburgh, and Washington University/St. Louis Children’s Hospital. Each site maintained their own institution-specific Institutional Review Board approval for the study. Additionally, approval from the Washington University Human Research Protection Office (HRPO) was acquired for the multicenter CSF repository and the analysis of CSF itself (Washington University (WU) HRPO #s 201203121). Screening and enrollment of patients at each site was performed by participating neurosurgeons and local HCRN coordinators using the following inclusion/exclusion criteria: *inclusion criteria*: preterm neonates ≤ 34 weeks estimated post-menstrual age (PMA) with birth weight < 1500 grams, Grade III or IV intraventricular hemorrhage (IVH), frontal occipital horn ratio (FOHR) ≥0.50, and >72 hour life expectancy, admitted to an HCRN Clinical Center prior to surgical intervention for PHH; *exclusion criteria*: primary caregiver refusing participation. Written consent was obtained from the parents or guardians of the children who served as subjects of the study. This study ran in parallel with (though lagged by 10 months) the HCRN study *Shunting Outcomes in Post-Hemorrhagic Hydrocephalus (SOPHH;* HRPO # 201105111*)* and thus followed standardized SOPHH parameters for PHH diagnosis and treatment, as previously described [1]. Of note, 11 subjects were enrolled at the lead site (WU) for the current study prior to the initiation of SOPHH; subject data points and CSF sample acquisition for these 11 subjects were identical to subjects enrolled through SOPHH.

### Acquisition of biospecimens

CSF was collected in the operating room at time of temporizing neurosurgical procedure (RES, VSGS, or hybrid RES/VSGS) following a standardized protocol (S1 File). Briefly, CSF was obtained directly from the ventricular catheter and distributed into screw-top microcentrifuge tubes labeled with an HCRN subject ID number for linkage to subject data in the HCRN Core Data Project. The CSF sample was then immediately transported on ice to a -80°C freezer for storage. Every 4–6 months, each site shipped their CSF samples on dry ice to the HCRN CSF Repository housed at the WU Tissue Procurement Core, where the samples were stored at -80°C until experimental analysis. This standardized protocol for CSF sample acquisition, preparation, processing, shipping, and storage allowed for consistency across HCRN sites, where clinical and laboratory facilities were not always co-localized. Thus, while ready accesss to centrifuges was limited at some sites, -80°C freezers were available at all centers, allowing freezing of CSF samples immediately on acquisition. This streamlined protocol also enabled acquisition of CSF samples in the evenings and on weekends, when many such cases are performed. Protocol compliance was recorded; two samples were removed from the analysis due to protocol irregularities. For comparison purposes, 16 CSF samples from age-matched preterm infants with no known neurological injury were acquired from the WU pediatric CSF repository (HRPO #201203126). The latter samples, considered Controls, were acquired via lumbar puncture for sepsis evaluation where the cultures remained negative.

### CSF protein measurements

Commercially available Enzyme-Linked ImmunoSorbent Assays (ELISAs) were used to measure the concentration of CSF APP, NCAM-1, L1CAM. Sandwich Duoset ELISA development systems (R&D Systems, catalog #DY-850, and DY-2408 respectively; Minneapolis, MN) were used to measure APP and NCAM-1 as previously described [28]. L1CAM levels were measured using a commercially available kit (DRG, catalog #EIA5074; Mountainside, NJ), also described previously [31]. Although the CSF matrix is complex, these ELISA kits have been used previously by our group and others to measure these specific analytes in CSF and in PHH and other, non-hemorrhagic hydrocephalus etiologies [30–34]. While possible that the CSF matrix could affect the performance of the ELISAs, our group has previously validated these measurements against Western blots and MS/MS proteomics, where PHH samples and protein-spiked samples were analyzed [28]. In all instances, ELISAs were run according to the manufacturer’s protocol. Briefly, plates were coated with a primary capture antibody and blocked prior to incubation with CSF. A secondary antibody was added, followed by streptavidin-HRP and tetramethylbenzidine. The ensuing chemical reaction was then stopped with sulfuric acid. The plates were washed between each of the steps except the final one. All CSF samples were run in duplicate, and the 96-well plates were read at 450nm on a Versamax microplate reader (Molecular Devices; Sunnyvale, CA). Protein concentrations were then determined using a four-parameter logistic standard curve as detailed by the manufacturer. Total CSF protein (TP) was estimated using the Pierce Bicinchoninic Acid protein assay kit (Thermo Scientific; Waltham, MA) according to the manufacturer’s protocol and as described previously [31]. Provided bovine serum albumin standards and CSF samples were placed into microplate wells in duplicate; the working reagent was then added and the plate was incubated at 37°C for 30 minutes. The plate was then cooled to room temperature and the absorbance was measured at 562nm on a plate reader. Total CSF protein levels were measured using a four-parameter logistic standard curve. In order to confirm that cellular lysate/debris was not impacting ELISA results, CSF protein concentrations from 10 PHH age- and ventricular-size matched CSF samples collected at the lead site and processed by centrifugation at 1000g/2500 rpm for 6 minutes were compared with the cohort of 23 PHH CSF samples from the lead site HCRN cohort (no centrifugation). No differences were noted in any of CSF proteins between the cohort of samples that had been centrifuged prior to freezing and storage versus those in the lead site HCRN cohort: NCAM-1 (474±132 ng/ml vs 469±301 ng/ml, p = 0.4797), APP (2794±1785 ng/ml vs 2172±1430 ng/ml, p = 0.1478), L1CAM (541±448 ng/ml vs 445±307 ng/ml, p = 0.2369), or TP (3101±1992 ug/ml vs 3870±4035 ug/ml, p = 0.2866).

### Neurodevelopmental testing

Trained psychometricians performed Bayley Scales of Infant Development-III (Bayley-III) testing at 15–30 months corrected age. Bayley-III is a standard, validated testing paradigm to assess neurobehavioral development in infants and toddlers [35]. The cognitive subtest assesses sensorimotor development, object exploration, manipulation, and relatedness, memory and concept formation, while the motor subtest assess fine and gross motor function, and the language subtest assesses receptive and expressive language development. Composite scores were derived from sums of the subtest scaled categories and used to compare the subject’s performance with age-matched, typically developing children.

Across the entire study cohort (n = 124), 31.5% (39/124) of subjects had Grade III IVH, while 68.5% (85/124) had Grade IV IVH; 41% (16/39) of subjects with Grade III IVH and 44.7% (38/85) of those with Grade IV IVH underwent for Bayley testing (p = 0.69).

### Statistical analysis

Continuous variables are summarized as mean ± standard deviation. Categorical variables are shown using counts and percentages. The relationships between CSF sample type (control vs PHH) and CSF protein concentrations were assessed using the Wilcoxon rank-sum test. Spearman’s correlation coefficient was used to assess relationships between FOHR and CSF protein concentrations. Pearson partial correlation coefficient, adjusting for PMA at birth and IVH grade, was used to analyze the relationship between CSF protein values and Bayley-III scores. Because there were a substantial number of statistical tests between the CSF proteins and Bayley-III scores, a linear setup method for controlling false discovery rate (FDR) was used to adjust the p-values [36]. As such, results were considered significant when both p<0.05 and Q (FDR) <0.20.

## Results

### Subject characteristics

A total of 124 subjects were enrolled in the current study across seven HCRN centers and 4 years (Table 1). The mean PMA at birth for the study cohort was 25.48 ± 2.08 weeks, while birthweight was 859.11 ± 250.64g (Table 2). Intraventricular hemorrhage was Grade III in 39 (31.5%) and Grade IV in 85 (68.6%). Of the 124 enrollees, 104 underwent temporizing treatment via implantation of RES (54, 51.9%), VSGS (44, 42.3%), or hybrid RES/VSGS (6, 5.8%) at a mean PMA of 31.00 ± 3.00 weeks. CSF samples were available from 98 of these temporizing procedures. Occipitofrontal circumference was 28.7 ± 3.98 cm at the time of temporizing intervention, while FOHR was 0.7 ± 0.07. At the time of data analysis, 119/124 subjects were eligible for Bayley-III testing, with 57 (48%) actually tested (Table 1). A total of 54 subjects who underwent Bayley-III testing had CSF samples from their temporizing procedure available for analysis.

**Table 1:** Subject data including temporizing surgeries, cerebrospinal fluid samples, and neurodevelopmental testing by Hydrocephalus Clinical Research Network site.

	Subjects Enrolled	Temporizing Procedure	CSF Sample Available	Eligible for Bayley-III	Bayley-III Completed
**HCRN Site**					
Site A	10	7	7	10	7
Site B	24	23	23	24	13
Site C	26	26	26	21	15
Site D	6	5	3	6	1
Site E	4	3	1	4	0
Site F	40	29	28	40	17
Site G	14	11	10	14	4
**Overall**	124	104	98	119	57

Temporizing procedures included ventricular reservoirs (RES), ventriculosubgaleal shunt (VSGS), or hybrid RES/VSGS devices. Of note, 54 subjects had both CSF samples and Bayley-III scores available for review. Bayley-III: Bayley Scales of Infant Development-III; CSF: cerebrospinal fluid; HCRN: Hydrocephalus Clinical Research Network.

**Table 2:** Surgical parameters for temporizing neurosurgical procedures for PHH across the Hydrocephalus Clinical Research Network.

	Overall
	(n = 104)
**Type of Temporizing Procedure**	
Ventriculo-subgaleal shunt	44 (42.3%)
Ventricular/Ommaya reservoir	54 (51.9%)
Hybrid RES/VSGS device	6 (5.8%)
**Post-menstrual age (weeks)**	31.0 ± 3.00
**Birthweight (g)**	859 ± 250.64
**Intraventricular Hemorrhage**	
Grade III	35 (33.7%)
Grade IV	69 (66.3%)
**Occipitofrontal circumference (cm)**	28.7 ± 3.98
**Frontal-Occipital Horn Ratio**^1^	0.7 ± 0.07

^1^Two subjects did not have pre-operative imaging available for analysis, therefore no FOHR was measured in these subjects. Temporizing procedures included ventricular reservoirs (RES), ventriculosubgaleal shunt (VSGS), or hybrid RES/VSGS devices.

As noted above, 20/124 subjects did not receive a temporizing procedure, and instead underwent permanent CSF diversion procedure [8 shunt cases, 12 endoscopic third ventriculostomy ± choroid plexus cauterization (ETV±CPC) cases] at 40.97 ± 5.13 weeks PMA with FOHR = 0.66 ± 0.09. Surgical outcomes for those with temporizing procedures (conversion to shunt or ETV ± CPC) and those with permanent CSF diversion procedures (shunt malfunction or ETV±CPC failure, infection, additional procedures) are the subject of a separate, forthcoming study.

### Cerebrospinal fluid proteins as biomarkers of PHH

**Fig 1:**
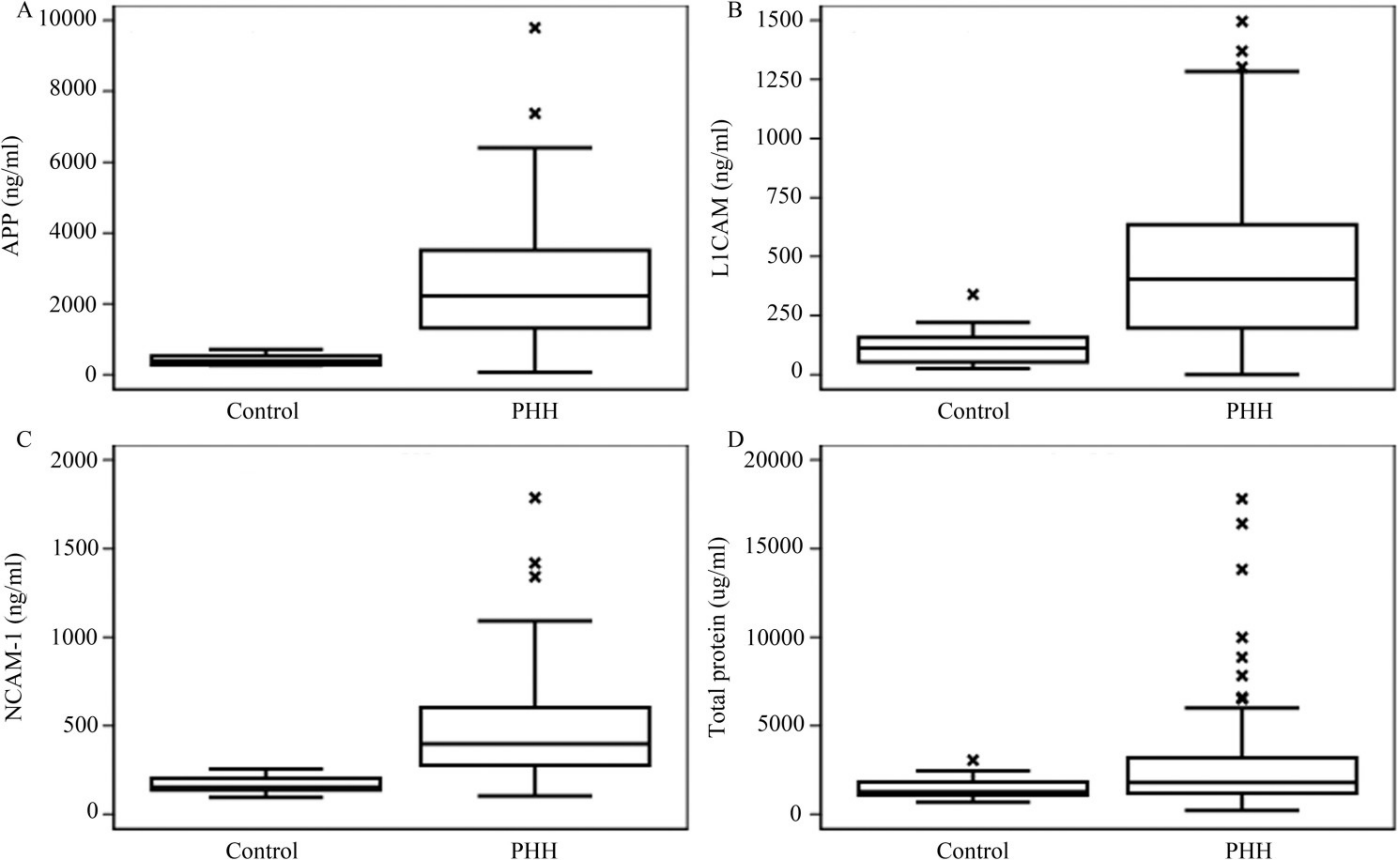
Cerebrospinal fluid amyloid precursor protein (APP), L1 cell adhesion molecule (L1CAM), neuronal cell adhesion molecule-1 (NCAM-1), and total protein (A–D respectively) are elevated at the time of initiation of post-hemorrhagic hydrocephalus treatment via temporizing neurosurgical procedure. For NCAM-1, in addition to the datapoints shown, there were outliers at 2653.49 and 7725.39 ng/ml.

**Fig 2:**
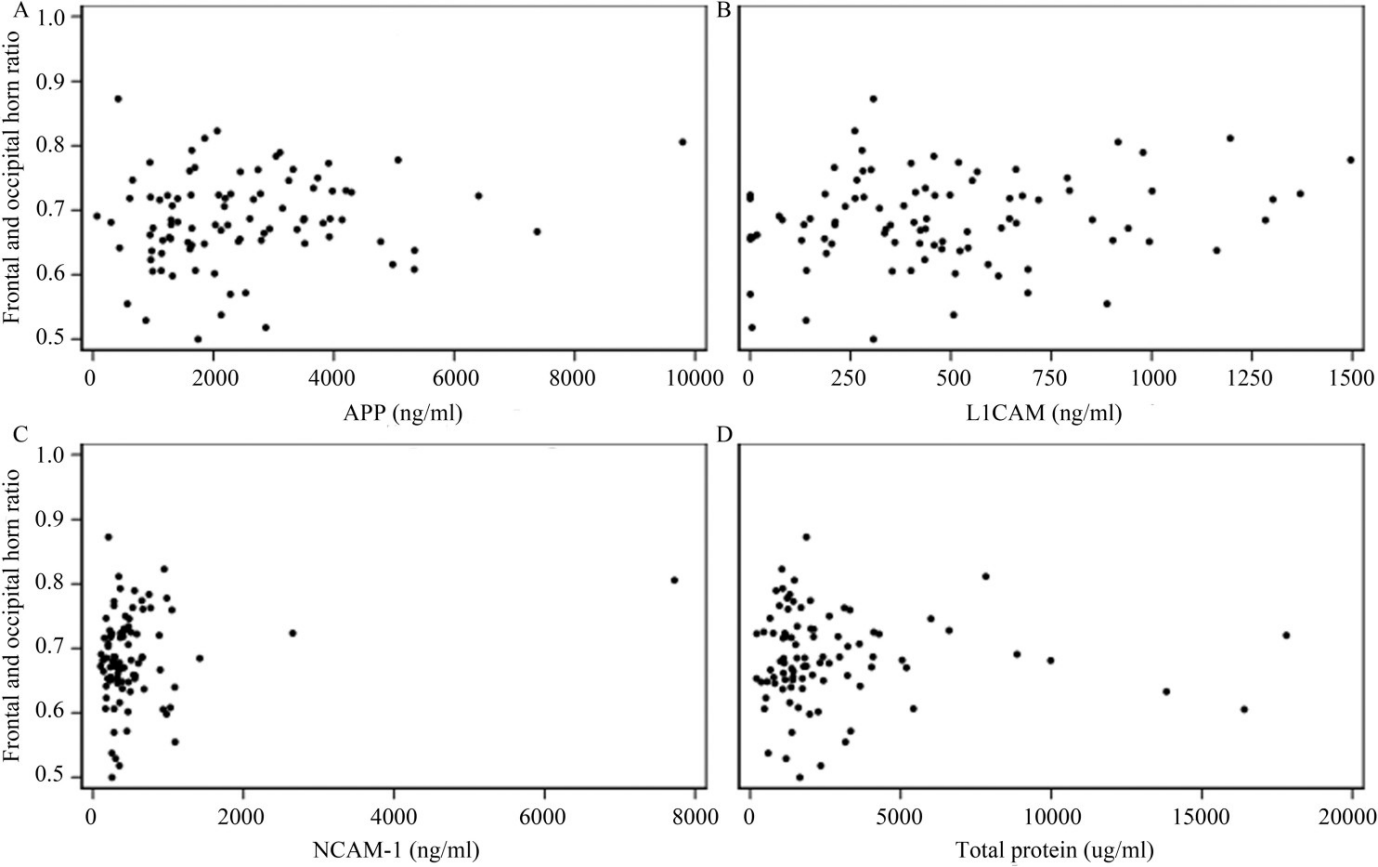
Cerebrospinal fluid amyloid precursor protein (APP), L1 cell adhesion molecule (L1CAM), neuronal cell adhesion molecule-1 (NCAM-1), and total protein (A–D respectively) were not associated with ventricular size in cross-sectional analysis.

CSF levels of APP, L1CAM, NCAM-1, and TP were measured in 16 controls and in 98 PHH subjects at the time of temporizing neurosurgical procedure. Each of the 3 specific CSF proteins and CSF TP were elevated at the time of the temporizing procedure (Fig 1A–1D). When comparing PHH associated with Grade III and Grade IV IVH, there were no differences in TP (388.7± 296.38 vs 562.3± 612.40, p = 0.506), APP (2538.7± 1592.45 vs 2418.0± 1646.45, p = 0.677), L1CAM (451.9±286.38 vs 490.4±366.52, p = 0.843), or NCAM-1 (465.8±287.60 vs 601.6±1028.06, p = 0.903). On cross-sectional analysis, no significant association was noted between FOHR and CSF APP, L1CAM, NCAM-1, or TP (Spearman correlation p-values of 0.102, 0.182, 0.177, 0.780, respectively; Fig 2A–2D).

### Relationship of CSF proteins to neurobehavioral outcome

Neurodevelopment was assessed using Bayley-III at 15–30 months corrected age in 54 subjects who had CSF collected at their temporizing neurosurgical procedures (21 with a subgaleal shunt, 32 with a reservoir, and 1 with a hybrid device) for PHH treatment across the HCRN (Tables 1 and 3). Notably, this study cohort contains a subgroup of subjects from SOPHH [1]; Bayley-III results for the entire SOPHH cohort will be reported elsewhere. Consistent with prior reports, the PHH infants in the current study exhibited poor Bayley-III results for all subtests, with the lowest scores observed for Grade IV IVH (Table 3). Of note, they did receive usual neurosurgical care as expected for infants with PHH [3, 18]. No significant relationship was identified between temporizing neurosurgical procedure type and Bayley-III cognitive, language, or motor scores. FOHR did exhibit a trend toward association with Bayley-III motor (p = 0.0647 and R = -0.2912) and cognitive scores (p = 0.0506 and R = -0.2966).

**Table 3:** Bayley Scales of Infant Development-III composite scores for study subjects with post-hemorrhagic hydrocephalus across the Hydrocephalus Clinical Research Network.

Bayley Scales of Infant Development-III Subtest	Composite Score		
	PHH	PHH	PHH
	IVH Grade III	IVH Grade IV	Total
	*n* = 16	*n* = 38	(*n* = 54)
Cognitive composite score	77.1 (19.15)	65.8 (11.51)	69.2 (14.96)
Language composite score	75.9 (20.23)	67.7 (16.66)	70.0 (17.89) ^#^
Motor composite score	74.9 (21.72)	57.5 (12.48)	62.3 (17.22)^#^

Of the 54 subjects with PHH, 16 had IVH Grade III prior to PHH, while 38 had IVH Grade IV prior to PHH. The Bayley-III scores shown are for PHH subjects and both IVH subcategories. Three subjects#, respectively, were unable to complete all phases of testing (2 pertaining to IVH Grade III and 1 pertaining to IVH Grade IV). Composites scores are reported as mean (standard deviation). For reference, composite scores in typically developing children are 100 for each domain.

Relationships between CSF APP, L1CAM, NCAM-1, and TP and Bayley-III composite scores are detailed in Table 4. Pearson partial correlation coefficients, adjusting for PMA at birth and IVH grade, were used to analyze the relationship between CSF protein values and Bayley-III scores. Notably, a highly significant relationship was identified between CSF NCAM-1 at temporization and Bayley-III motor score (Pearson partial correlation coefficient = -0.422, p = 0.007, FDR Q = 0.089). A more modest relationship between CSF NCAM-1 and Bayley-III cognition and language was also noted, but this marginally reached significance with rigorous correction for false discovery (cognition Pearson partial correlation coefficient = -0.335, p = 0.030, FDR Q = 0.182 and language Pearson partial correlation coefficient = -0.314, p = 0.048, FDR Q = 0.194). Based on the relationships between FOHR and Bayley-III scores noted above, the analyses of the NCAM-1 and Bayley-III cognitive and motor scores were adjusted for FOHR yielding Pearson partial correlation coefficient = -0.318, p = 0.042, FDR Q = 0.194 (cognitive) and Pearson partial correlation coefficient = -0.415, p = 0.01, FDR Q = 0.114 (motor). No significant relationships were observed between CSF APP, L1CAM, or TP and any Bayley-III parameter.

**Table 4:** Correlations between the Bayley-III cognitive, language, and motor composite scores and CSF concentration of APP, NCAM-1, L1CAM, and total protein at the time of temporizing neurosurgical procedure.

		APP	L1CAM	NCAM-1	Total Protein
**Bayley-III Cognitive**	Pearson partial Correlation	-0.082	-0.180	-0.335	0.021
	P-value	0.606	0.253	0.030	0.893
	Q-value	0.808	0.679	0.182	0.893
**Bayley-III Language**	Pearson partial Correlation	-0.047	-0.060	-0.314	-0.147
	P-value	0.772	0.714	0.048	0.365
	Q-value	0.843	0.843	0.194	0.679
**Bayley-III Motor**	Pearson partial Correlation	-0.095	-0.149	-0.422	-0.140
	P-value	0.567	0.367	0.007	0.396
	Q-value	0.808	0.679	0.089	0.679

## Discussion

In recognition of substantial neurobehavioral impairments in infants with PHH, there is increased focus on efforts directed at enhancing neurodevelopmental outcomes. One major factor impeding advances in clinical care and clinical research in PHH is a dearth of tools capable of providing real-time input for the clinical management of PHH, from establishing the diagnosis of PHH to the timing of intervention, monitoring of therapeutic efficacy, and evaluating the impact of PHH treatment on long-term neurodevelopmental outcome. Such biomarkers of PHH would also be of tremendous value in stratifying patients and estimating long-term outcomes in clinical trials designed to establish evidence-based management approaches for the care of infants with PHH. The current study aimed to address this critical void by examining candidate CSF biomarkers of neurodevelopment in PHH. Notably, CSF NCAM-1 demonstrated a highly significant association with Bayley-III motor score as well as more modest relationships with Bayley-III cognition and language. This is the first report of a CSF protein acquired at the time of intervention demonstrating an association with long-term neurodevelopment in PHH. No significant relationships were observed between the other CSF proteins studied and Bayley-III scores.

APP, L1CAM, and NCAM-1 were selected as candidate CSF biomarkers of PHH based on a body of work drawing on findings from a seminal proteomic analysis of PHH CSF [28]. Among protein mediators of neurodevelopment in the index proteomics study, APP demonstrated the greatest change in concentration, while L1CAM showed the highest statistical significance [28]. NCAM-1 demonstrated both robust responsiveness and high significance. Notably, all three of these candidate biomarkers have important roles in neurodevelopment, but have also been implicated in neurodevelopmental and/or neurodegenerative disorders. APP, for instance, is best known as a contributor to the pathophysiology of Alzheimer’s disease, but its primary role in brain physiology is likely in generating important neurotrophic factors (e.g. sAPPα) for normal neurodevelopment and neuroprotection [37]. L1CAM is essential in the migration of neural precursors, synaptogenesis, dendritic arborization, and corticogenesis [38, 39], and mutations in L1CAM are known to cause hydrocephalus, cerebral anomalies, spasticity, and developmental delay [40, 41]. Among other roles, the protein NCAM-1 regulates synaptic connectivity and cortical circuit formation, and aberrations in NCAM-1 have been implicated in neuropsychiatric diseases, including autism [42–46].

Cerebrospinal fluid APP, L1CAM, and NCAM-1 have been shown to correlate with ventricular size in individual human infants with PHH [30]. Measuring the levels of each of these biomarkers in serial CSF samples obtained from ventricular reservoirs in individual subjects, CSF APP, L1CAM, and NCAM-1 all tracked ultrasound-based measures of ventricular size (FOHR) in real time throughout the neonatal period, with CSF APP demonstrating the best correlation. Preliminary data in the same report suggested that NCAM-1 may also be correlated with intracranial pressure. These findings, coupled with the developmental relevance of each of these proteins, provided the scientific premise for selecting these three candidate CSF biomarkers of neurodevelopment in PHH.

The Bayley-III remains the gold standard for evaluating neurodevelopment in early childhood. The Bayley-III provides summary scores for cognition, expressive language, receptive language, fine motor skills, and gross motor skills. While the Bayley-III is a widely accepted tool for infants and young children, there are limitations in its application and interpretation, as several reports have suggested that the Bayley-III may underestimate developmental delay [47] and its fidelity in predicting long-term outcomes is debated [48]. Further work is underway in an ongoing HCRN randomized clinical trial (ClinicalTrials.gov Identifier: NCT04177914) to rigorously examine the relationship of CSF NCAM-1 and neurodevelopment, with formal neurobehavioral testing using additional tools for up to 5 years after hydrocephalus treatment.

In the current study, and similar to other recent reports in infant hydrocephalus, we observed low performance on the Bayley-III in our participants (Table 3) [3, 7, 18, 49–52]. Consistent low performance on Bayley-III may reflect limitations in the responsiveness of this tool in PHH, where neurological impairments may be substantial, but it also may cause a ‘floor effect’ and affect our ability to detect robust associations between CSF biomarkers and long-term neurodevelopmental impairments in this population. Notably, such limitations in our current tools provide excellent justification for additional biomarkers with enhanced responsiveness and long-term predictive capabilities.

Relatedly, neurodevelopmental outcomes in PHH, whether measured using Bayley-III, as in this study, or other tools administered at later ages, would be expected to be affected by myriad factors, not the least of which are those related to the IVH itself (age at the time of hemorrhage, size, grade, location, and laterality of hemorrhage) [3–5, 27, 53–63] and the development of PHH requiring surgical treatment [3]. The arc of neurosurgical treatment [temporizing treatments (RES/VSGS/hybrid; CSF volume removed), permanent treatments (shunt/ETV-CPC), shunt infection, and the number and severity of shunt malfunctions/revisions] would also be expected to impact outcomes and are the subject a forthcoming study. Beyond IVH and PHH, other acute or chronic newborn medical illnesses, including sepsis or other infection, chronic lung disease, nutrition, and visual or auditory deficits, also may affect neurodevelopment. Similarly, access to physical, occupational, and other therapies would be expected to affect neurodevelopmental trajectories. Due to sample size considerations, the potential impact of these factors on Bayley-III composite scores was not accounted for in the current study.

Similar to previous studies [4, 5, 27, 63], our results demonstrate higher levels of neurological disability in PHH associated with Grade IV IVH compared with Grade III IVH. In order to account for this difference in our analyses, we employed Pearson partial correlation coefficients, adjusting for PMA at birth and IVH grade. As differences in follow-up rates in Grade III versus Grade IV IVH subjects could potentially introduce bias into our results, we also examined Bayley-III testing in these two groups and found no difference in the return rate for Bayley-III testing (p = 0.69).

Our group and others [7, 9, 64] have demonstrated that larger ventricle size at the time of neurosurgical intervention may be associated with worse neurodevelopmental outcome, though this remains controversial and is an area of active study [18, 64–66]. Notably, in the current study, the FOHR at temporization was high (0.7 ± 0.07), indicating substantial ventriculomegaly prior to intervention. These values significantly exceed the ‘late’ criteria in the randomized trial of early versus late ventricular intervention study (“ELVIS”) [8] and may have resulted in a ‘ceiling effect,’ limiting our ability to detect relationships among CSF biomarkers and FOHR and attenuating the strength of associations that we observed between CSF biomarkers and outcomes (e.g. R = -0.415 for CSF NCAM-1 and Bayley-III motor score). Indeed, the Bayley-III scores reported herein are lower than those reported in ELVIS and a few other studies[4, 5, 63, 67, 68], though comparison to other studies is difficult due to variation in Bayley testing (Bayley-II versus Bayley-III) or incomplete or aggregated data [4, 63].

The CSF samples included in this study were acquired prospectively from 7 different HCRN sites across North America and aggregated at the HCRN PHH CSF Repository at Washington University. The multi-institutional nature of this repository is both a significant strength and a limitation. Despite standard operating procedures, there was likely inherent variability in the handling, processing, storage, and shipping of CSF samples across sites. This variability enhances the generalizability of our results but also attenuates our ability to detect and quantify statistical relationships. To address the differences in access to research facilities across HCRN sites, our protocol specified for freezing CSF samples immediately after acquisition. While our quality control analysis identified no differences in the concentrations of NCAM-1, APP, L1CAM, or TP between these HCRN samples and samples centrifuged immediatedly after acqusition (see Methods above), it remains possible that cellular lysate or debris could have impacted our CSF protein analyses. Comparison of lumbar and ventricular CSF also creates a potential confound owing to possible rostro-caudal CSF protein gradients. We have addressed this thoroughly in prior reports [31, 34], one of which confirmed PHH-associated increases over control in CSF APP, L1CAM, and NCAM-1 when analyzing only lumbar CSF samples (i.e. control and PHH CSF samples were all acquired via lumbar puncture). Further, there may be bias introduced by the variability in enrollment or selection of study participants across the HCRN or the modest Bayley-III testing rate (47.9%). These limitations relate to variability among HCRN centers in terms of location, practice environment, patient population, access to Bayley-III assessments, and practical limitations in obtaining these assessments (long-distance travel, complex chronic illnesses). As previously detailed in a comprehensive review [34], myriad other candidate CSF-based biomarkers have been proposed for IVH/PHH (and hydrocephalus of other etiologies) for predicting clinical course and understanding the basic mechanisms underlying the pathophysiology of IVH/PHH and associated developmental consequences. Finally, a separate study investigating the association of CSF biomarkers with surgical parameters (device malfunction, treatment failure, infection, etc.) is forthcoming.

## Supporting information

S1 File Hydrocephalus Clinical Research Network cerebrospinal fluid repository: Procedures for acquisition, storage, and shipping of biospecimens. (PDF)

## List of abbreviations

APP: amyloid precursor protein
Bayley-III: Bayley Scales of Infant Development-III
CPC: choroid plexus cauterization
CSF: cerebrospinal fluid
ELISA: enzyme-linked immunosorbent assay
ETV: endoscopic third ventriculostomy
FDR: false discovery rate
FOHR: frontal-occipital horn ratio
HCRN: Hydrocephalus Clinical Research Network
HRPO: Human Research Protection Office
IVH: intraventricular hemorrhage
L1CAM: L1 cell adhesion molecule
NCAM-1: neural cell adhesion molecule 1
PHH: post-hemorrhagic hydrocephalus
PMA: post-menstrual age
RES: ventricular reservoir
SOPHH: Shunting Outcomes in Post-Hemorrhagic Hydrocephalus
TP: total protein
VSGS: ventriculo-subgaleal shunt
WU: Washington University
