# Supplementary material for: Cerebrospinal fluid NCAM-1 concentration is associated with neurodevelopmental outcome in post-hemorrhagic hydrocephalus of prematurity
Source: PLoS One. 2021 Mar 10;16(3):e0247749. doi: 10.1371/journal.pone.0247749 (PMC7946285; doi:10.1371/journal.pone.0247749)
Supplement: S1 File — (PDF) [file pone.0247749.s001.pdf]

Hydrocephalus Clinical Research Network Cerebrospinal Fluid Repository

Procedures for Acquisition, Storage, and Shipping of Biospecimens

Updated April 2, 2019 from the 2011 HCRN Procedures and
Adapted From International Consensus Guidelines for Biobanking of Cerebrospinal Fluid[1-4]

Collection of CSF Samples

Cerebrospinal fluid (CSF) will be collected in the operating room at time of surgery for temporizing device (ventricular reservoir, ventriculosubgaleal shunt), CSF shunt implantation, or endoscopic third ventriculostomy (ETV) ± choroid plexus cauterization (CPC). For each procedure, 5 ml of CSF will be obtained directly from the ventricular catheter or endoscope. The first 2 ml should be sent for routine clinical studies (CSF cell counts, protein, glucose, and, if indicated, gram stain/culture) or expunged if no clinical studies are obtained. The OR scrub nurse will then distribute the remaining 3 ml the CSF into 3 separate 1.5 ml screw-top polypropylene microcentrifuge tubes using a clean, dry, sterile syringe (1 ml/tube). The scrub nurse will then close the tubes and hand them off the operative field to the circulating nurse or the research coordinator. The research coordinator will take the CSF samples, apply the labels provided to each tube, and place the tubes on ice. The subject ID number and a number corresponding to the type and date of the surgical procedure will be recorded in permanent ink on the labels. Samples will be transferred on ice and stored in a -80°C clinical or laboratory freezer until shipment to Washington University. (Temporary storage in a -20°C freezer is permitted if the auto-defrost function is set to ‘off.’)

CSF Sample Inventory

An inventory of PHH CSF samples will be maintained in an Excel file and in the Essential Documents Binder at each institution. Inventories should contain the following information:

- subject ID
- Date of surgery
- Volume of CSF and number of microcentrifuge tubes
- Time of acquisition of CSF (draw time)
- Time CSF samples were placed in the freezer (freeze time)

In order to clarify which CSF samples are awaiting shipment to Washington University, the electronic Excel inventory file should contain tabs/worksheets for both “Active Inventory” and “Shipped Samples.”

Scheduled Shipments

CSF samples will be shipped to the Washington University Tissue Procurement Core (WUTPC) Facility on scheduled dates twice a year.

Shipment Agreements and Procedures

Material Transfer Agreements: In order to share tangible resources such as CSF or other biological specimens, institutions require a Material Transfer Agreement (MTA). An MTA details the protections, rights, and obligations of both parties/institutions. Confidentiality, publication rights, ownership and licensing rights of any intellectual property, limitations on use of the requested materials, liability and indemnification are addressed in an MTA. The Washington

University Office of Technology Management (OTM) reviews, negotiates, and approves all MTAs. Each MTA is different and must be reviewed on a case-by-case and institution-by-institution basis. Please refer to Appendix II for an example of incoming MTA at Washington University. Each site will be responsible for obtaining and presenting the MTA to Washington University for review, though the Washington University HCRN Site P.I. and Coordinator will facilitate this.

Other Regulatory Components: Shipping biological substances, formerly *diagnostic specimens* or *patient specimens*, in the United States or internationally is regulated under either hazardous materials regulations (U.S.) or dangerous goods regulations (international). In order to ship PHH CSF samples, research coordinators must first undergo training in Department of Transportation (DOT)/International Air Transport Association (IATA) shipping practices. DOT/IATA training is typically administered via brief online educational module by each institution's Environmental Health and Safety (EHS) division. For the purposes of shipping, PHH CSF samples are considered "specimens without pathogens" and are thus not subject to IATA regulations. The person completing the shipping air bill will serve as the "designated shipper" and a copy of the shipping documentation should be sent to the local institution's EHS prior to transport, and these records should be retained for 2 years.

Shipping Requirements: Shipping materials must be of appropriate quality to withstand leakage of sample contents, vibration or shocks, changes in atmospheric pressure or humidity, mechanical handling associated with ordinary transportation. Essentially, even if there is a leak of the contents of the primary receptacle (microcentrifuge tubes), there should be no leakage to the outside of the shipping container. Shipping of biological substances requires *triple packaging*. The first level of packaging (primary receptacle) is the watertight screw-top microcentrifuge tube. Microcentrifuge tubes, with labels attached, will be placed in a cryogenic shipping box and sealed in a watertight biohazard pressure bag (second level of packaging) that also contains a 10x10 cm square absorbant pad, which serves the dual purposes of dampening impact and absorbing the CSF if any of the microcentrifuge tubes leak. A sturdy Styrofoam cooler and custom-fit outermost cardboard box will be provided to serve as the third level of packaging.

Standard Procedure for Shipping CSF:
1. Shipping will take place on a scheduled Tuesday twice a year. On the day prior to shipping, construct an itemized list (from Excel inventory file) showing subject ID number, number of samples for each subject, the type and date of samples for inclusion in the shipping package.
2. Use lab coats, gloves and protective eyewear when handling the specimens.
3. On the day of shipping, remove the microcentrifuge tubes from the storage freezer and verify that the labels remain legible and attached to the appropriate tube. Verify that the screw top is tight. Cover screw top with Parafilm® (provided).
4. Place each microcentrifuge tube into the cryogenic shipping box. Place the lid over the tubes securing them into the box. Tape the box to secure the lid. Place the box in a biohazard bag with the absorbant pad. Make sure to securely seal the biohazard bag.
5. Place the biohazard bag(s) (containing the microcentrifuge tubes) in to the Styrofoam cooler (provided) and fill the cooler with dry ice. Place the Styrofoam cooler lid onto the cooler, but do not tape the lid into place. (Dry ice shipments must not be sealed with an airtight seal--packages must allow for the release of carbon dioxide gas.)
6. Place the itemized list of samples being shipped as well as the Tissue Procurement Core Biospecimen Submission Form (and any laboratory logs) into a watertight ZipLock® bag. Include the courier waybill number on the list and seal the bag. The ZipLock® bag with the documentation must be placed on the lid of the cooler .
7. Put the cooler in the outermost cardboard box and seal the box securely with packing

tape.

8. Apply the following labels to the outside of the cardboard box:

   - Name, address and telephone number of the shipper
   - Name, address and telephone number of the consignee:
   - Label: “Exempt human specimens”
   - Label: Class 9 miscellaneous hazardous materials
   - Labels: Two “Up” arrows, placed on opposite sides of the box

9. Arrange for pick-up by FedEx (drop boxes cannot be used). Make sure billing information is correct.
10. Notify recipient of the shipment. Include the following information: FedEx tracking number, name of contact person, name of site submitting the samples. An electronic version of the inventory submitted should be included in the notification email for each shipment.

Receipt, Accession, and Storage of PHH CSF Samples

In most cases, shipments will arrive to the Washington University Tissue Procurement Core (WUTPC) facility in the morning of the Wednesday post shipment. On arrival, the package will be opened and cooler removed. The itemized inventory list will be reconciled with CSF samples received, and the samples will be accessioned into caTissue with a limited amount of subject information (subject ID, date of CSF sample, total volume of samples, number of microcentrifuge tubes), and bar codes will be applied directly to the microcentrifuge tubes. Once accessioned and bar coded, CSF samples will be stored at -80°C in a WUTPC facility laboratory freezer

Retrieval of Samples for Experimental Analysis

In preparation for experimental analysis, the WUTPC inventory will be queried using caTissue, and an online specimen request form will be submitted. CSF samples will be retrieved from the WUTPC for analysis. In general, only one 1.5 ml microcentrifuge tube will be requested from any given subject at in a single request. The samples will be thawed, spun at 2000g for 10 minutes, and the supernatant and aliquotted immediately for experimental analysis or further storage. In cases where cell preservation is desired for RNA isolation, samples will be spun at 400g for 10 minutes. In either case, the pellet will be frozen and stored. After experimentation, any unused portion of the sample will be frozen and stored at -80°C in the Limbrick laboratory rather than returning it to the WUTPC.

Procedures for maintaining confidentiality

CSF samples will be shipped and accessioned with subject ID number only. All patient identifiers will be removed from the sample. The subject ID key will be maintained securely at the institution of origin only. Relevant clinical data will be accessed when necessary through the Core Data Project. Data collected in this project will not include names, but some identifying information (such as date of birth, gender, and date of surgery) will be recorded. Project investigators will protect the confidentiality of the research data in accordance with privacy regulations such as HIPAA. The following measures will be taken to preserve confidentiality:

- All data (paper or electronic) will be maintained in secure locations in locked offices.
- The patient log containing the patient's identifying information and unique identifying number will be stored in a secure locked location at the originating site.
- Data Coordinating Center (DCC) staff work under strict confidentiality agreements.

- All analyses and reports will be presented in aggregate fashion.
- No identifiable data, either patient, surgeon or site-specific, will be released by at any time.
- All research publications and presentations arising from this study will be presented in aggregate form, and at no time during or after the study will any research data be released with patient, surgeon or center identifiable fields.
- Security in data transmission is maintained with 128-bit SSL data encryption.

References

1. Teunissen EC et al. A consensus protocol for the standardization of cerebrospinal fluid collection and banking. *Neurology* 73:1914-1922, 2009.
2. Teunissen CE et al. Biobanking of CSF: International standardization to optimize biomarker development. *Clinical Biochemistry* 47:288-292, 2014.
3. Willemse EAJ and Teunissen CE. Biobanking of Cerebrospinal Fluid for Biomarker Analysis in Neurological Diseases. Karimi-Busheri F (ed.), *Biobanking in the 21st Century, Advances in Experimental Medicine and Biology*, 864, 79-93, 2015.
4. Tashjian RS et al. Biobanking of Cerebrospinal Fluid. William H. Young (ed.), *Biobanking: Methods and Protocols, Methods in Molecular Biology*, vol. 1897, 107114, 2019.

Appendix II. Incoming MTA for Washington University

Washington University in St. Louis
CENTER OF TECHNOLOGY MANAGEMENT

MATERIAL TRANSFER QUESTIONNAIRE FORM FOR ***RECEIVING*** MATERIALS FROM A NON-PROFIT RESEARCH INSTITUTION

Submit this form each time to receive materials from a non-profit research institution.

Receiving WU Investigator: ________________________ Campus Box: __________

Department: ____

Shipping Address:____

Phone: __________________ Fax : _________________________ E-mail: __________________

Additional Department Contact Person:________________ Phone:_______________

Fed Ex Account No:__________________________

Material Requested:____

Provider (Organization): ____________________________ Contact:_____________________

Address: ____

City:__________________________State:_______Zip:_____________________

Phone: ____________________ Fax: _________________E-mail: _________________________

- Is the research being done *in vivo* or *in vitro*? __________Please provide Animal Studies protocol number if *in vivo*:____________.
- Will the material be used in conjunction with other material(s) being provided under another MTA? Yes ____ No ____ If yes, please identify provider: _____________________________________

Appendix II (cont.)

- Identify ALL sources of funding for the research project(s) in which you plan on using the requested materials. Please note both commercial and non-commercial sponsors (if only using department funds, please indicate):

- In this research, will you or any co-investigators likely create a new substance or derivative that contains or incorporates the requested materials? Yes_____ No___
- Will you explore a new use for the material? YES___NO___

Signature of W.U. Investigator________________________ Date___________

Please return this questionnaire via e-mail to Carl Morrell at the Office of Technology Management, Campus Box 8013, e-mail: morrellc@otm.wustl.edu , Ph: 747-0922, Fax: 362-5872.

***To assist us in expediting your request, please include any correspondence with and agreements from the provider, preferably in an electronic format.**

Appendix III. Check List for Shipping PHH CSF samples.

Initial	Item/Activity
	Microcentrifuge tubes labelled with HCRN subject # and date of sample
	Microcentrifuge tube screw-on caps are tight and wrapped with Parafilm®
	Microcentrifuge tubes are wrapped in paper towel and placed individually in biohazard bags
	Biohazard bags placed into cooler, and cooler filled with dry ice.
	Itemized list of contents with courier waybill number in ZipLock® bag within the cooler
	Cooler placed into outermost box and sealed with packing tape.
	Outermost cardboard box sealed with packing tape and labelled with:<br>Name, address and telephone number of the shipper<br>“Exempt human specimens”<br>“Class 9 miscellaneous hazardous materials:<br>Two “Up” arrows, placed on opposite sides of the box<br>Name, address and telephone number of the consignee:
	Arrange for pick-up by FedEx (drop boxes cannot be used).

Adapted from Center for Disease Control website documents.
